# Physiological changes in post-hatchling green turtles (*Chelonia mydas*) following short-term fasting: implications for release protocols

**DOI:** 10.1093/conphys/coz016

**Published:** 2019-05-16

**Authors:** Duane T March, Ellen Ariel, Suzy Munns, Donna Rudd, David Blyde, Les Christidis, Brendan P Kelaher

**Affiliations:** 1 National Marine Science Centre, School of Environment, Science and Engineering, Southern Cross University, Coffs Harbour, NSW, Australia; 2 College of Public Health, Medical and Veterinary Sciences, James Cook University, Townsville, QLD, Australia; 3 Veterinary Department, Sea World, Gold Coast, QLD, Australia

**Keywords:** Catabolic, fasting, fitness, post-hatchling, release

## Abstract

Relocation of sea turtle nests and the retention of post-hatchlings for head-starting programs are both commonly used to improve conservation outcomes and facilitate eco-tourism ventures. Currently, there is little literature surrounding the husbandry protocols required during these programs to optimize post-release outcomes. To assess the impact of varied feeding regimes on exercise performance, (which will hereafter be referred to as ‘fitness’), 40 10-month-old captive post-hatchling green turtles (*Chelonia mydas*) were divided into four groups of 10 and fasted for either 3, 9, 10 or 15 h. The animals were then subjected to a fitness test via repetitive use of the ‘righting reflex’ on land. Health assessments were conducted prior to the fitness test, including; heart rate, haematocrit (Hct), heterophil to lymphocyte ratio and the measurement of 11 biochemical analytes, including pH, partial pressures of carbon dioxide (P_v_CO_2_) and oxygen (P_v_O_2_), lactate, bicarbonate (HCO_3_^−^), sodium (Na^+^), potassium (K^+^), chloride (Cl^−^), ionized calcium (iCa^2+^), glucose and urea. Results were corrected for multiple comparisons and significant differences among groups were demonstrated for temperature, pH, HCO_3_^−^, iCa^2+^, urea and lactate. To investigate physiological relationships between analytes, correlation coefficients were calculated between fitness and glucose, fitness and lactate, glucose and lactate, pH and iCa^2+^, pH and K^+^, pH and P_v_CO_2_, pH and HCO_3_^−^ and Hct and K^+^. Following correction for multiple comparisons, significant relationships were seen between pH and iCa^2+^ and pH and HCO_3_^−^. Post-hatchling turtles appear to enter a catabolic state when exposed to short-term fasting. While this did not have a direct impact on fitness, the production of an intense energetic output from a catabolic state may induce a physiological debt. This study suggests that handling that induces a physical response should be minimized and animals should be fed within 10 h of release.

## Teaser

Post-hatchling green turtles were subject to varied fasting regimes followed by a fitness assessment. Blood was collected prior to this assessment and biochemical changes indicated that short-term fasting induced a catabolic state, but did not compromise exercise output. Facilities holding these animals should consider this when designing pre-release protocols.

## Introduction

The management of post-hatchling sea turtles in captive environments is becoming increasingly common. This is due to both conservation strategies, where relocation is used as an effective strategy to decrease hatchling mortality and assist with population management in areas of high tidal inundation (Dutton *et al.*, 2005; Wyneken *et al.*, 1988) and, more recently, to facilitate eco-tourist–based ventures (Tisdell and Wilson, 2005). Extensive research has determined the factors that are required to optimize the success of nest relocation including not moving the eggs between 12 h and day 21 of gestation (Harry and Limpus, 1989; Limpus *et al.*, 1979; Parmenter, 1980), minimizing movement during transport (Limpus *et al.*, 1979), maintaining optimal incubation temperatures (Burgess *et al.*, 2006; Fisher *et al.*, 2014) and controlling moisture content (McGehee, 1990), and new research is examining the efficacy of delaying embryonic development prior to movement via the creation of hypoxicx conditions (Williamson *et al.*, 2017). The process of hatching and emergence can take several days (Fisher *et al.*, 2014; Godfrey and Mrosovsky, 1997), and following this, the hatchlings can be successfully released from the beach adjacent to the nest location. The ‘swim frenzy’ is then utilized to propel them through predator-rich near shore environments to access the required, deeper offshore currents (Wyneken and Salmon, 1992).

However, in sea turtle hatcheries such as those located in Sri Lanka (Tisdell and Wilson, 2005), Indonesia (Haryati *et al.*, 2016) and Brazil (de Vasconcellos Pegas and Stronza, 2010), the release dates for animals can be planned to coincide with peak tourist numbers, resulting in hatchlings that can remain in care for months prior to release (Tisdell and Wilson, 2005). Prolonged retention of hatchlings may compromise a turtle’s chance of survival post-release (Pilcher and Enderby, 2001), and therefore, maintenance of appropriate husbandry regimes and the employment of optimal pre-release processes are required for successful post-release outcomes. Digestion and fasting can both alter physiological processes and feeding regimes and should be considered in the release plan. Adult sea turtles have unique physiological adaptions to deal with the extended periods of fasting that occur during the nesting period (Perrault *et al.*, 2016; Perrault *et al.*, 2014), including changes in hormone levels associated with appetite control (Goldberg *et al.*, 2013) and the mobilization of fat stores to provide the energy required to complete the nesting process (Hamann *et al.*, 2002; Perrault and Stacy, 2018; Plot *et al.*, 2013). Physiological tolerance to short-term fasting has also been documented in sub-adult animals, where a decrease in blood glucose was not detected until animals were fasted for 1 week (Moon *et al.*, 1997). Digestion has been shown to induce a postprandial metabolic alkalosis in other reptiles (Hicks and Bennett, 2004) and an increase in metabolic rate called the ‘specific dynamic action’ (SDA) (Secor, 2009). The SDA of captive post-hatchlings is relatively small compared to large, carnivorous reptiles, with the upregulation of the metabolic rate to facilitate digestion lasting up to 4 h post-feeding (Kowalski, 2005). The impact of digestion and fasting on post-hatchling exercise output is unknown, and hence it is difficult to predict how each process may affect post-release outcomes.

Metabolic studies in wildlife have become increasingly feasible with the advent of hand-held point of care blood analysers, such as the VetScan i-STAT®. (Alberghina *et al.*, 2015; Hunt *et al.*, 2016; Perrault *et al.*, 2018; Stoot *et al.*, 2014; Wolf *et al.*, 2008). These machines are capable of assessing blood gases and plasma analytes and have the potential to provide near real-time insights into the metabolic state of an animal. Here, we assess the impacts of short-term fasting on exercise performance, which will be referred to in this study as ‘fitness’ and use a hand-held analyser to detect the associated physiological changes in blood analytes, in post-hatchling green turtles.

## Materials and methods

Housed at James Cook University following collection from Heron Island in Southern Queensland, Australia (government permit WITK15765815; Animals Ethics Committee Permit Number A2309) were 40 green turtle hatchlings, 10 months prior to the following experimental protocols. The animals were collected during an afternoon emergence, held overnight and then transported to the research facility the following day, arriving ~30 h after collection. Hatchlings were housed in individual sea water tanks and were fasted for 9 days to allow the yolk sac to be utilized. Following this, food was introduced slowly over a 2-week period until the animals were eating 5% of their body mass per day with a diet that composed of one-third fish/prawn meat, one-third commercial fish pellets and one-third mixed green vegetables. All of the ingredients were finely diced and bonded with gelatin and a multivitamin supplement (SeaTabs B.T.F.S, Pacific Research Labs, Ramona, CA) and were frozen prior to feeding. Adjustments were made fortnightly based on weight to maintain feeding at 5% body mass per day. At the time of the experiment, the mean (± standard deviation) body mass of the post-hatchlings was 129.6 ± 21.9 g. Each day, the tanks housing the animals were placed in direct sunlight for 1 h to provide ultraviolet (UV) exposure. To avoid the introduction of movement stress associated with transferring the animals outside, on the 2 days of the experiment, the animals were not placed outside for routine UV supplementation. The tank temperatures were allowed to equilibrate to ambient temperatures, and the tank temperature for each animal was recorded immediately prior to the health assessment.

The turtles were divided into 4 groups of 10 and were subjected to either a 3-, 9-, 10- or 15-h fasting period; a health assessment (described below) was conducted prior to the fitness test. The time intervals were selected to include both the presence and absence of the SDA but were also limited due to logistical constraints. Given the pelagic nature of these animals at this life stage, swim tests were attempted to measure fitness; however, in response to current, the animals would passively drift. Hence, to assess fitness, all animals were placed in dorsal recumbency to encourage the ‘righting reflex’, which is a commonly used measure of hatchling fitness (Booth *et al.*, 2013; Maulany *et al.*, 2012; Wood *et al.*, 2014) and provided a consistent fitness challenge among groups for this experiment. To measure fitness, the number of times the animal successfully turned over, within a maximum period of 3 min or until the animals remained in dorsal recumbency for a period in excess of 20 s, was recorded.

Data collected during health assessments included heart rate and analysis of blood health analytes. Heart rate was measured using an ultrasound machine (GE Logiq Book) in B-mode using a curve-linear probe positioned on the centre of the plastron at a frequency of 5 MHz, while the animal was held in an upright position. Heart beats were counted for 30 s and multiplied by 2 to calculate the beats per minute. Collected from the dorsal occipital sinus was 150 μL of venous blood using a 25-gauge, 1.6 cm needle and a 1-mL syringe following aseptic preparation of the skin based on previously described techniques (Owens and Ruiz, 1980), and the sample was carefully observed during collection to monitor for lymphatic contamination. A blood smear was prepared immediately with fresh blood and stained with Diff Quik (Point of Care Diagnostics Healthcare, Artarmon, NSW) to enable the heterophil to lymphocyte ratio to be calculated using standardized methods (Fudge, 2000). The remaining sample was transferred to a 1-mL BD Microtainer® tube (Beckton Dickson, Franklin Lakes, NJ) containing lithium heparin. Biochemical analysis of the heparinized blood was conducted immediately using a VetScan i-STAT®1 System (Analyzer Model 300A; Abaxis, Union City, CA, USA). CG4+ (ABBT-03P85–25) cartridges were used to analyse pH, partial pressures of carbon dioxide (P_v_CO_2,_) and oxygen (P_v_O_2_)_,_ lactate and bicarbonate (HCO_3_^−^). Chem8+ (ABBT-09P31–25) cartridges were used to measure sodium (Na^+^), potassium (K^+^), chloride (Cl^−^), ionized calcium (iCa^2+^), glucose, urea and haematocrit (Hct). The CG4+ analysis was conducted before the Chem8+ and the time between analyses was minimized to avoid temporal changes. The total volume of blood used in the sample collection did not exceed the recommended maximum amount for chelonian species of 0.8% of body weight (Mans, 2008).

The i-STAT® machine performs analysis of samples at 37°C, and hence the following parameters were temperature corrected _(TC)_ as per previous studies in sea turtles (Harms *et al.*, 2003; Keller *et al.*, 2012), where ΔT = 37°C—tank temperature. Data were corrected to the temperature of the tank, as opposed to the body temperature of the animal. While a degree of endothermy has been documented in sub-adult and adult green turtles, with deep body temperatures ranging from 2.2 ± 0.2°C to 1.7 ± 0.2°C above ambient temperatures at 20°C and 30°C, respectively (Heath and McGinnis, 1980), tank temperature was selected given the potential trauma involved in obtaining a deep body temperature in animals with small cloaca’s.

pH_ TC_ = pH _37°C_ + (0.014/°C) × ΔT) (Kraus and Jackson, 1980).

P_v_CO_2 TC_ = P_v_CO_2 37°C_ × (10^−0.019 ΔT^) (Ashwood *et al.*, 1983).

P_v_O_2 TC_ = P_v_O_2 37°C_ × (10^−0.0058 ΔT^) (Ashwood *et al.*, 1983).

HCO_3_^−^ was calculated using the Henderson–Hasselbalch equation HCO_3_^−^ = αCO_2_ × P_v_CO_2_ × 10^(pH-pka)^, where αCO_2_ and pKa were calculated using the equations described by Stabenau and Heming (1993) and P_v_CO_2 TC_ and pH _TC_^.^

iCa^2+^_TC_ = iCa^2+^ (1 + 0.53(pH–pH _TC_)) (Fogh-Andersen, 1981).

The mean ± standard error was calculated for each analyte, including the mass of the animals in each group and the data were assessed for homogeneity of variance and normality of distribution using Levene’s test and the Shapiro–Wilk test, respectively. To determine differences among groups of varied fasting intervals, where data met these assumptions, a one-way analysis of variance (ANOVA) for each analyte was performed. Temperature, heart rate, fitness outcomes, P_v_O_2_, lactate, glucose and K^+^ did not meet these assumptions, and hence a Kruskal–Wallis analysis was used. Given the potential for inflated type 1 error due to multiple comparisons, the Holm–Bonferroni sequential correction method (Abdi, 2010; Holm, 1979) was also used. To assess for pairwise differences between groups where significant differences were detected, Bonferroni and Dunn’s *post hoc* analyses were used for ANOVA and the Kruskal–Wallis results, respectively. Pearson correlation coefficients (*r*) were calculated for hypotheses about the relationships among analytes and fitness; however, given multiple correlations increased the chance of type 1 error, only select variables were assessed, and correction for multiple comparisons was conducted via the Bonferroni method (Curtin and Schulz, 1998). All analyses were conducted on IBM SPSS Statistics Version 24 Licence 9.6.0.0.

## Results

**Figure 1:**
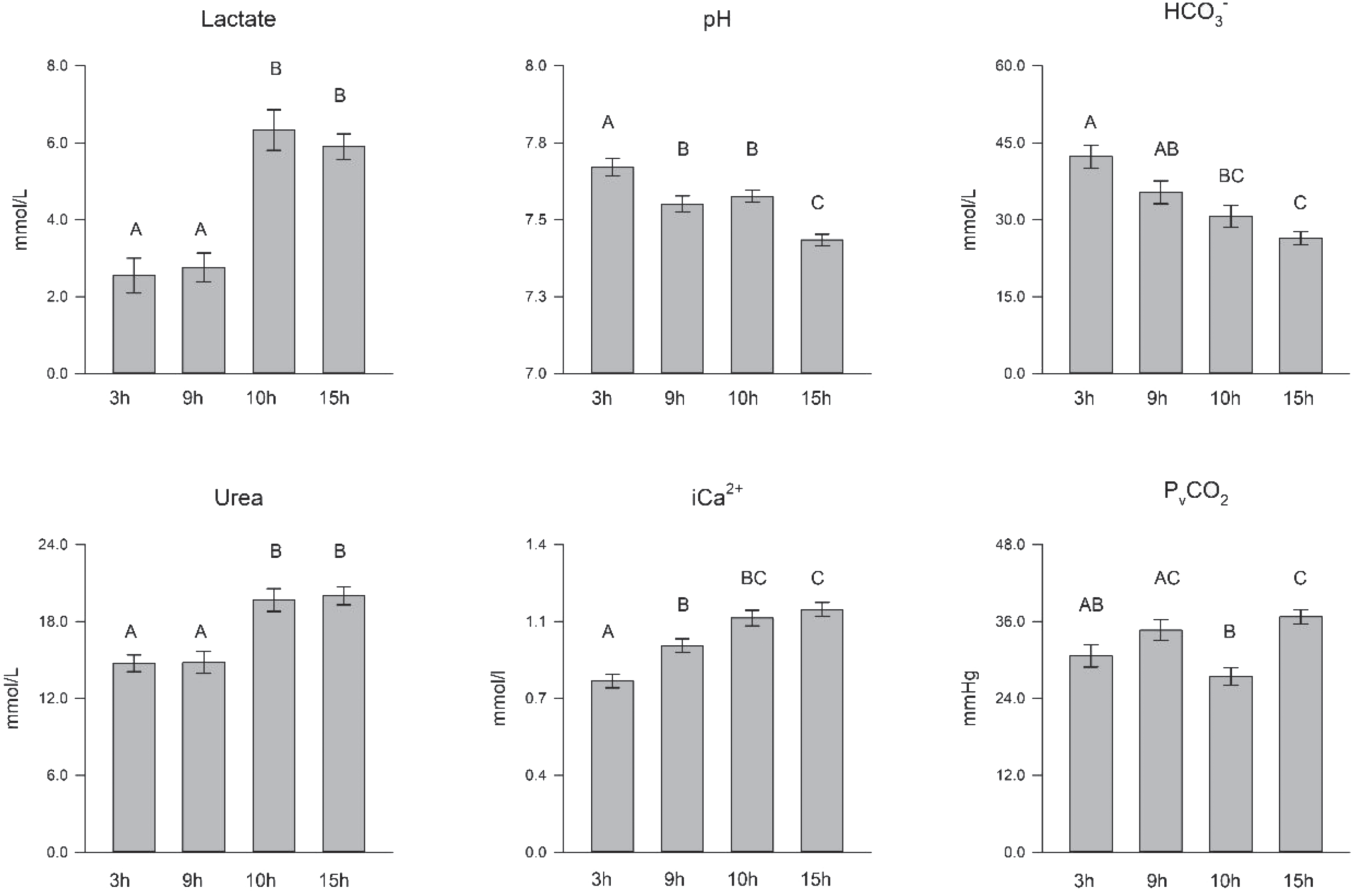
Mean and standard error for groups at 3, 9, 10 and 15 h after feeding for analytes where significant differences were detected with letters denoting pairwise differences.

The median and range of the analytes for each group, along with an indication of the analytes that failed Levene’s test for homogeneity of variance and the groups that failed the Shapiro–Wilk test for normality, are presented in Table 1. Also presented in Table 1 is the fact that for 12 data points, K^+^ levels were below the detectable limit. Where this occurred, a nominal value equal to the lowest detection point, 2 mmol/L, was assigned. Analytes that were calculated to be significantly different among groups following correction are shown in Fig. 1. Significant differences were calculated for temperature (*P* < 0.01), lactate (*P* < 0.01), pH **(***P* < 0.01), HCO_3_^−^ (*P* < 0.01), urea (*P* < 0.01), iCa^2+^ (*P* < 0.01) and P_v_CO_2_ (*P* < 0.01).

**Table 1:** Median and *range* of blood analytes, temperature and heart rate for groups fasted for 3, 9 10 and 15 h

	**3 h**		**9 h**		**10 h**		**15 h**	
Weight (g)	129.5	*(98.0–164.0)*	128.0	*(104.0–164.0)*	124.0	*(96.0–156.0)*	134.6	*(83.0–169.0)*
Fitness^¥^	8.5	*(5.0–12.0)*	6.0	*(4.0–15.0)*	5.0	*(2.0–7.0)*	8.0	*(3.0–9.0)*
Temperature^¥^ (°C)	24.2	*(23.5–25.3)*	26.1	*(25.9–26.3)*	23.8	*(23.6–26.4)*	30.5	*(30.3–30.9)*
Hct (%)	20.0	*(17.0–23.0)*	21.0	*(19.0–27.0)*	19.0	*(14.0–23.0)*	18.0	*(13.0–22.0)*
Heart Rate^¥^	23.5	*(10.0–47.0)*	45.0	*(12.0–50.0)*	39.0	*(20.0–54.0)*	41.0	*(16.0–58.0)*
H:L	0.52	*(0.12–1.1)*	0.48	*(0.29–0.89)*	0.73	*(0.42–1.5)*	0.48^†^	*(0.37–1.2)*
Glucose^¥^ (mg/dL)	111.6	*(102.6–117.0)*	120.6	*(95.4–136.8)*	124.2	*(102.6–172.8)*	122.4^†^	*(113.4–145.8)*
Lactate (mmol/L)	2.1	*(1.1–4.8)*	2.7	*(1.0–5.0)*	5.8	*(5.1–10.5)*	5.9	*(4.3–8.1)*
pH	7.64	*(7.59–7.85)*	7.55	*(7.44–7.68)*	7.59	*(7.45–7.66)*	7.43	*(7.34–7.55)*
HCO_3_^−^ (mmol/L)	41.6	*(34.5–56.0)*	33.9	*(21.9–47.0)*	27.9	*(22.0–43.0)*	25	*(22.0–34.6)*
Urea (mmol/L)	14.6	*(11.3–18.2)*	14.9	*(10.1–18.8)*	19.7	*(13.4–23.5)*	20.4	*(15.8–23.9)*
iCa^2+^ (mmol/L)	0.78	*(0.64–0.92)*	0.91	*(0.78–1.11)*	1.03	*(0.86–1.18)*	1.10	*(0.95–1.29)*
P_v_CO_2_ (mmHg)	31.0	*(18.9–37.4)*	34.9	*(28.4–41.4)*	26.3	*(21.4–32.6)*	37.7	*(21.8 42.0)*
P_v_O_2_ (mmHg)	55.4	*(47.7–94.6)*	51.4	*(43.2–60.6)*	59.2	*(47.3–73.7)*	56.3	*(42.1–59.5)*
Na^+^ (mmol/L)	144.0	*(139.0–147.0)*	141.0	*(139.0–145.0)*	145.0	*(141.0–148)*	144.5	*(139–148.0)*
Cl^−^ (mmol/L)	100.0	*(88.0–105.0)*	101.0	*(93.0–109.0)*	102.0	*(91.0–106.0)*	103.0	*(96.0–107.0)*
K^+^ (mmol/L)	2.2	*(2.0–2.6)*	2.6	*(2.0–2.9)*	2.1	*(2.0–2.4)*	2.4^†^	*(2.2–3.0)*

Hct = haematocrit, ^*^K^+^= 12 values were below detectable limit, P_v_CO_2_ = partial pressure carbon dioxide, Na^+^ = sodium, iCa^2+^ = ionized calcium, HCO_3_^−^ = bicarbonate.

¥ ^¥^Analyte failed Levene’s test for homogeneity of variance between groups

^†^Analyte failed the Shapiro–Wilk test for normality of distribution within the group.

Pairwise analysis showed there was no significant difference in the temperature of the holding tanks between animals fasted for 3 h compared to 9 h (*P* = 0.08) or 10 h (*P* > 0.99) or between animals fasted for 9 h compared to 10 h (*P* = 0.29) or 15 h (*P* = 0.11). Significant differences in holding tank temperatures were calculated between groups fasted for 3 h compared to 15 h (*P* < 0.01) and between animals fasted for 10 h compared to 15 h (*P* < 0.01). There was no significant difference in lactate between animals fasted for 3 h and 9 h (*P* > 0.99) or animals fasted for 10 h compared to 15 h (*P* > 0.99). There was a significant difference between animals fasted for 3 h compared to animals fasted for 10 h (*P* < 0.01) and 15 h (*P* < 0.01) and between animals fasted for 9 h compared to animals fasted for 10 h (*P* < 0.01) and 15 h (*P* < 0.01). The pH declined significantly during the postprandial period, and significant differences were calculated among all groups with the exception of animals fasted for 9 h compared to animals fasted for 10 h (*P* > 0.99). A similar postprandial decline was observed with HCO_3_^−^ with significant differences measured when animals were fasted for 3 h were compared to animals that were fasted for 10 h (*P* < 0.01) and 15 h (*P* < 0.01). There was no significant difference in urea between animals fasted for 3 h and 9 h (*P* > 0.99) or animals fasted for 10 h compared to 15 h (*P* > 0.99). There was a significant difference between animals fasted for 3 h compared to animals fasted for 10 hs (*P* < 0.01) and 15 h (*P* < 0.01) and between animals fasted for 9 h compared to animals fasted for 10 h (*P* < 0.01) and 15 h (*P* < 0.01). iCa^2+^ increased during the postprandial interval with significant differences calculated between animals fasted for 3 h compared to animals fasted for 9 h (*P* < 0.01), 10 h (*P* < 0.01) and 15 h (*P* < 0.01) and between animals fasted for 9 h compared to animals fasted for 15 h (*P* < 0.01). However, there was no significant difference between animals fasted for 9 h compared to animals fasted for 10 h (*P* > 0.05) or animals fasted for 10 h compared to 15 h (*P* > 0.99). Significant differences in P_v_CO_2_ levels were calculated for animals that were fasted for 10 h compared to 9 h (*P* < 0.01) and 15 h (*P* < 0.01).

The results of the Pearson’s correlation assessment can be seen in Table 2. Following adjustment of the alpha value via the Bonferroni correction technique, there was a significant negative correlation between pH and iCa^2+^ (*r* = −0.58, *P* < 0.01) and a significant positive correlation between pH and HCO_3_^−^ (*r* = 0.77, *P* < 0.01); however, no significant correlations were calculated within groups.

**Table 2:** Pearson’s correlation coefficient (*r*) and the associated *P* value for selected analytes within groups

	**Overall**		**3 h**		**9 h**		**10 h**		**15 h**	
	**r**	***P***	**r**	***P***	**r**	***P***	**r**	***P***	**r**	***P***
Fitness: glucose	−0.17	0.29	−0.15	0.70	0.12	0.75	0.07	0.85	−0.08	0.82
Fitness: lactate	−0.16	0.34	0.25	0.52	0.42	0.23	−0.13	0.72	0.72	0.02
Glucose: lactate	0.34	0.03	−0.71	0.03	0.36	0.30	−0.03	0.93	−0.10	0.79
pH: iCa^2+^	−0.58	<0.01^*^	−0.24	0.53	−0.24	0.53	0.17	0.66	−0.47	0.20
pH: K^+^	−0.35	0.04	0.51	0.17	−0.21	0.58	0.67	0.04	−0.28	0.47
pH: P_v_CO_2_	−0.37	0.03	−0.22	0.56	−0.23	0.56	−0.60	0.10	−0.03	0.95
pH: HCO_3_^-^	0.77	<0.01^*^	0.42	0.26	0.71	0.03	0.38	0.28	0.79	0.01
Hct: K^+^	0.23	0.18	0.31	0.41	0.50	0.17	0.19	0.63	−0.33	0.39

Hct = haematocrit, K^+^ = potassium, P_v_CO_2_ = partial pressure carbon dioxide, iCa^2+^ = ionized calcium, HCO_3_^−^ = bicarbonate.

* ^*^Denotes significant correlation following Holm–Bonferroni correction.

## Discussion

The mean values for glucose, Cl^−^ and, Na^+^ fall into the local reference intervals for green turtles (Flint *et al.*, 2010). K^+^ levels were lower than these reference intervals and levels recorded in other studies on different species of sub-adult sea turtles (Hunt *et al.*, 2016; Innis *et al.*, 2014). While hypokalemia can be associated with metabolic alkalosis (Campbell, 2006), no significant correlation with pH was calculated, and hence the low readings may reflect decreased dietary intake. The P_v_CO_2_ is similar to previous studies using point-of-care analysers in sea turtles; however, the P_v_O_2_ was lower (Hunt *et al.*, 2016). Tissue hypoxia can be related to a functional deficiency of mature red blood cells (Hodges *et al.*, 2007), and while the Hct was within the normal reference intervals for sub-adult and adult green sea turtles in Australia, it would be considered low compared to studies conducted in sub-adult animals in the northern hemisphere (Bolten and Bjorndal, 1992; Page-Karjian *et al.*, 2014; Stacy *et al.*, 2018) and is lower than previously recorded values in leatherback hatchlings (Reina *et al.*, 2002). Hct levels can be impacted by iatrogenic error, such as lymphatic contamination or sample hemolysis. If the former were to occur, based on the comparatively low levels of K^+^ in lymphatic fluid compared to plasma (Thrall *et al.*, 2012), a positive correlation would be seen between the two analytes. This is in contrast to hemolysis, where the destruction of the cell would liberate the intracellular K^+^ and elevate K^+^ levels (Yücel and Dalva, 1992) in the analysed sample, while the cellular destruction would decrease the Hct. This process would generate a negative correlation between the two analytes. However, there was no significant correlation calculated between Hct and K^+^, and hence neither hypothesis adequately explains the results. Previous studies utilizing the i-STAT machine in fish have shown reduced Hct readings relative to microcapillary controls (Harter *et al.*, 2014). The proposed explanation for this, which may be relevant to sea turtles, is that the algorithm used to calculate Hct is based on a non-nucleated, spherical red blood cell, as opposed to the elliptical, nucleated red blood cells that fish and reptiles have and hence is not as accurate when used in these species (Harrenstien *et al.*, 2005).

The pH declined postprandially for green turtles in two distinct phases. The initial decline between 3 and 9 h may be reflective of a waning metabolic alkalosis that accompanies digestion (Hicks and Bennett, 2004) and is supported by decreasing HCO_3_^−^ levels during this period. This timeline is consistent with the SDA calculated for post-hatchling green turtles fed 2% of their bodyweight, where a peak increase occurred 1 h post-ingestion, with VO_2_ returning to near normal levels by 4 h (Kowalski, 2005). The decline in pH that occurs between animals fasted for 10 h compared to animals fasted for 15 h may be the result of an accumulation of acidic by-products produced via catabolic metabolism, as increases in the ketone body β-hydroxybutyrate have been documented previously in sub-adult green turtles following fasting (Price *et al.*, 2013). The steady rise in iCa^2+^ postprandially and the significant negative correlation with pH are reflective of this acidifying state, as H^+^ can displace iCa^2+^ from their binding sites, increasing serum levels (Wang *et al.*, 2002).

The lactate levels observed in the early postprandial stages are typical of hatchlings at rest, while the later stages of fasting are more consistent with levels following anaerobic exercise (Hamann *et al.*, 2007), indicating a shift in these animals towards a state of catabolic metabolism. The increased lactate levels likely occurred secondary to the liberation of pyruvate from skeletal muscle following the onset of glycogenolysis (Rui, 2014) that allows lactate to be used directly by skeletal muscles as a gluconeogenic substrate (Gleeson, 1996). The hypothesis of metabolic transition to catabolism is also supported by the elevation in urea in fasted animals, as elevated levels of urea have been recorded during fasting in nesting hawksbill (Goldberg *et al.*, 2013) and leatherback turtles (Plot *et al.*, 2013), as amino acids are catabolized from muscle protein. The decrease in P_v_CO_2_ calculated at 10 h may be a compensatory mechanism associated with this metabolic transition, as hyperventilation can be used to compensate for metabolic acidosis (Hartzler *et al.*, 2006).

The temperatures of the holding tanks were measured immediately prior to blood collection to provide the information needed to temperature correct the i-STAT data. Significant variations in pre-assessment temperature among groups represent a potential confounding factor in this study. To address this, analysis of covariance (ANCOVA) analysis was the preferred method of analysis, with temperature as the covariate; however, many analytes displayed heterogeneous regression slopes, which precluded the use of this analysis technique. There was no significant difference between the mean temperature of animals fasted for 3 h compared to animals fasted for 10 h or 9 h compared to 15 h, and yet significant differences were observed between these groups for a number of analytes, supporting the hypothesis that physiological changes were induced via varied postprandial periods independent of temperature variations.

Despite the elevated VO_2_ that is seen during the SDA to facilitate digestion in reptiles that have consumed large, high protein meals, these animals are also able to mobilize metabolic resources to facilitate exercise during this period (Bennett and Hicks, 2001; Secor *et al.*, 2000). Post-hatchling green turtles generally employ anaerobic metabolic pathways to produce the energy required to perform intense exercise (Hamann *et al.*, 2007), and hence repeat analysis after the fitness test would have been of interest to assess the physiological changes induced by animals that were already engaged in a state of catabolic metabolism. The ability of turtles fasted for 15 h to perform an equal number of turnovers as the turtles fasted for 3 h suggests that this is possible. Unfortunately, repeat analysis was not conducted due to the small size of the animals, limiting the volume of blood available for analysis.

Short-term fasting appears to induce a catabolic state of metabolism in post-hatchling green turtles; however this does not appear to directly impact upon the ability of the animal to produce physical activity. While these animals appear to be able to physiologically compensate for this process, they may incur a metabolic debt that the animal will need to repay once caloric input and pathways of aerobic metabolism are reinitiated. As more intensive management options are employed to improve the conservation of sea turtles, both the immediate and medium-term fitness of these animals post-release need to be considered. Further work investigating energetics in post-hatchlings, including the time to physiologically recover from intense exercise in both fasted and fed animals, is required before best practice regimes can be employed in release protocols.
